# Osteoid Osteoma of the Distal Humerus Mimicking Sequela of Pediatric Supracondylar Fracture: Arthroscopic Resection—Case Report and A Literature Review

**DOI:** 10.1155/2013/247328

**Published:** 2013-03-04

**Authors:** Jordi Font Segura, Sergi Barrera-Ochoa, Albert Gargallo-Margarit, Eva Correa-Vázquez, Anna Isart-Torruella, Xavier Mir Bullo

**Affiliations:** ^1^Hand Surgery Unit, Hospital Universitari USP Dexeus, Spain; ^2^Hospital Universitari Vall d'Hebron, Universitat Autònoma de Barcelona, Spain

## Abstract

Osteoid osteoma (OO) is a small and painful benign osteoblastic tumour located preferentially in the shaft of long bones near the metaphyseal junctions, with a predilection for the lower limbs. Juxta- and intra-articular OOs are rare and even though hip, elbow, and talus are the most commonly reported locations, they may be found in any joint accounting for approximately 13% of all osteoid osteomas. There is usually a significant time delay between symptom initiation and diagnosis when the lesion is present in an uncommon location due to the diagnostic challenge it presents due to the lack of classical clinical signs and/or radiographic features found in the extra-articular lesions. A case of a distal humerus OO of a 15-year-old girl is presented to point out that a confounding factor, such as a previous paediatric supracondylar fracture, may further delay the already difficult diagnosis of a juxta- or intra-articular osteoid osteoma and also to emphasize the possibility of arthroscopic treatment of such lesions.

## 1. Introduction

Osteoid osteoma (OO) is a small and painful benign osteoblastic tumour described for the first time as a separate entity by Jaffe in 1935 [1]. It is located preferentially in the shaft of long bones near the metaphyseal junctions, with a predilection for the lower limbs [2]. Juxta- and intra-articular OOs are rare and even though they may be found in any joint, hip, elbow, and talus are the ones most reported in the literature [3–5]. They account for approximately 13% of all osteoid osteomas [6]. The occurrence of OO in uncommon locations presents a diagnostic challenge and usually there is significant time delay between symptom initiation and diagnosis due to the lack of clinical signs and radiographic features that exist in the well-established classical hallmarks of an extra-articular lesion [2–4, 7, 8]. In treating intra-articular OO, no differences have been reported between using open or an arthroscopic approach [6, 9]. 

We present our case hoping to be alerted that a confounding factor, such as a previous paediatric supracondylar fracture, may delay even more the already difficult diagnosis of a juxta- or intra-articular osteoid osteoma and also to emphasized the possibility of arthroscopic treatment of such lesions.

## 2. Case Report

A 15-year-old girl was referred to our hospital due to on-going pain and restricted range of motion of her left elbow. The symptoms began 2 years ago and she had already undergone conservative treatment for a 12-month period in the initial centre from where she was referred. Her medical history revealed a paediatric supracondylar fracture (Garland Stage III) when she was 7 years old, which was treated by closed reduction and fixation with two pins inserted from the lateral side of the elbow.

On physical examination, she complained of persistent pain in the elbow joint, sometimes radiating down to the forearm. It was more intense at night and subsided partially with salicylates. A limitation of the flexion-extension arc was present (100°/−5°) but both pronation and supination were preserved. The patient reported that even though she had not achieved full extension after the supracondylar fracture, the range of elbow motion had gotten worse over the past 2 years.

**Figure 1:**
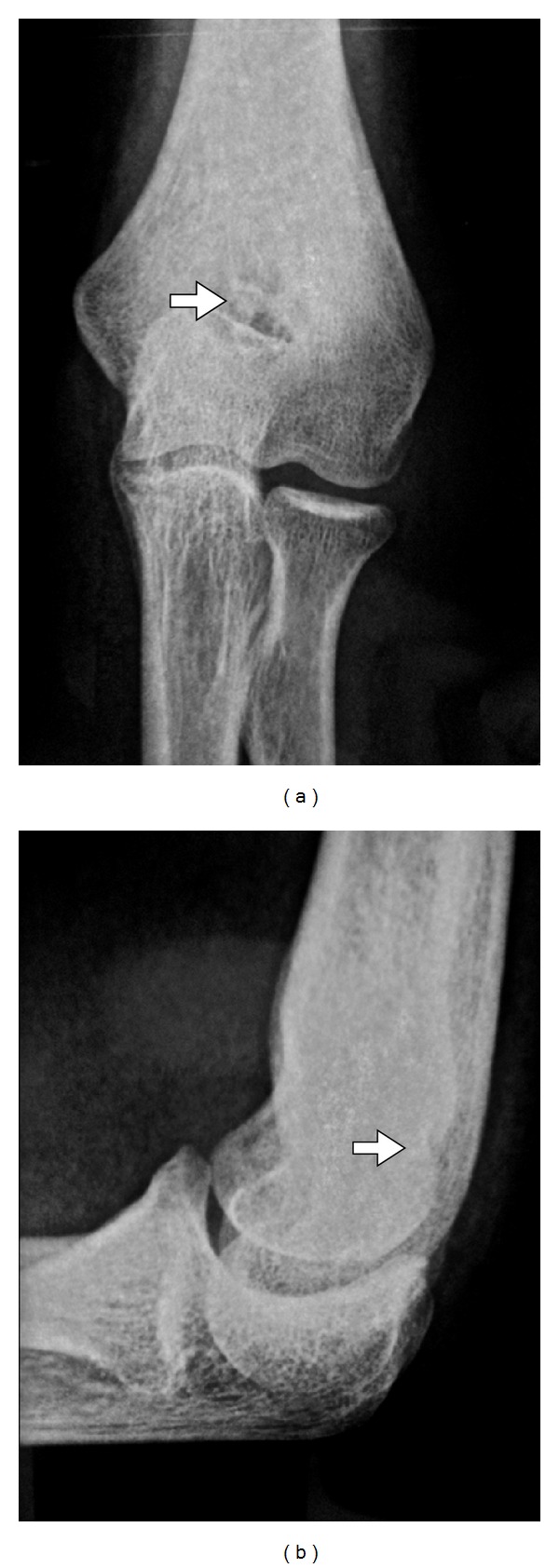
AP/lateral plain radiograph: a nidus in olecranon fossa humeri with small central sclerosis surrounded by reactive sclerosis.

**Figure 2:**
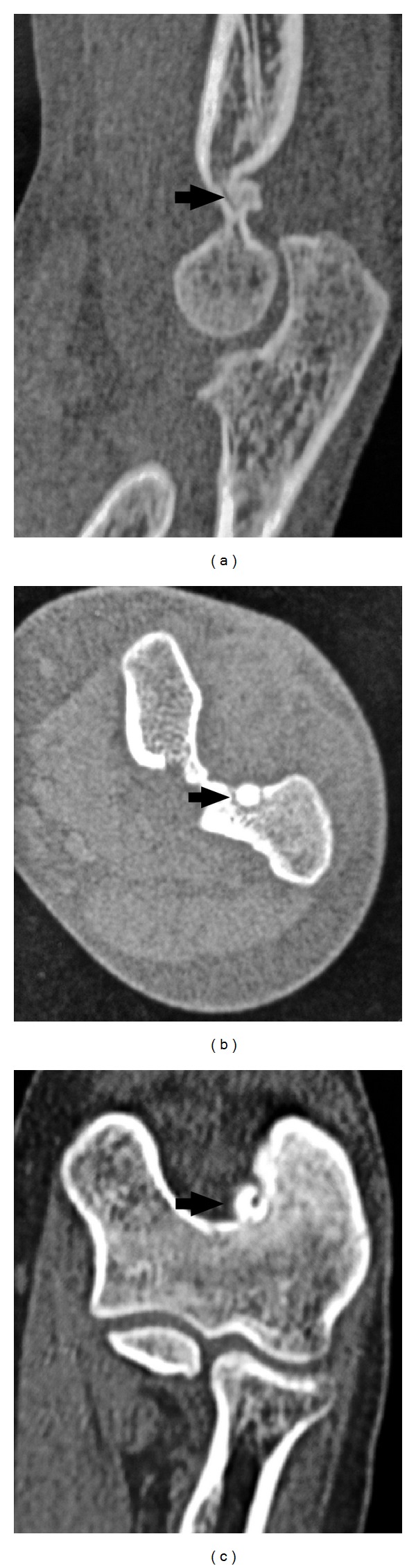
Sagittal, axial, and coronal computed tomography sections of the elbow show a 7 mm diameter cystic lesion that respects the cortical bone and is located at the cartilage-cortical bone junction in the posterolateral part of the olecranon fossa.

**Figure 3:**
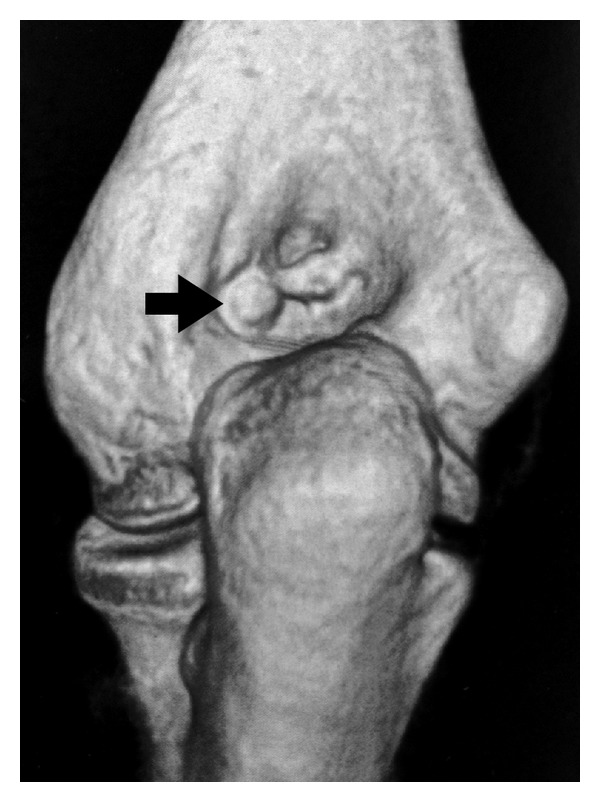
CT image reconstruction of the elbow: protrusion of a nidus in the lateral side of the olecranon fossa.

**Figure 4:**
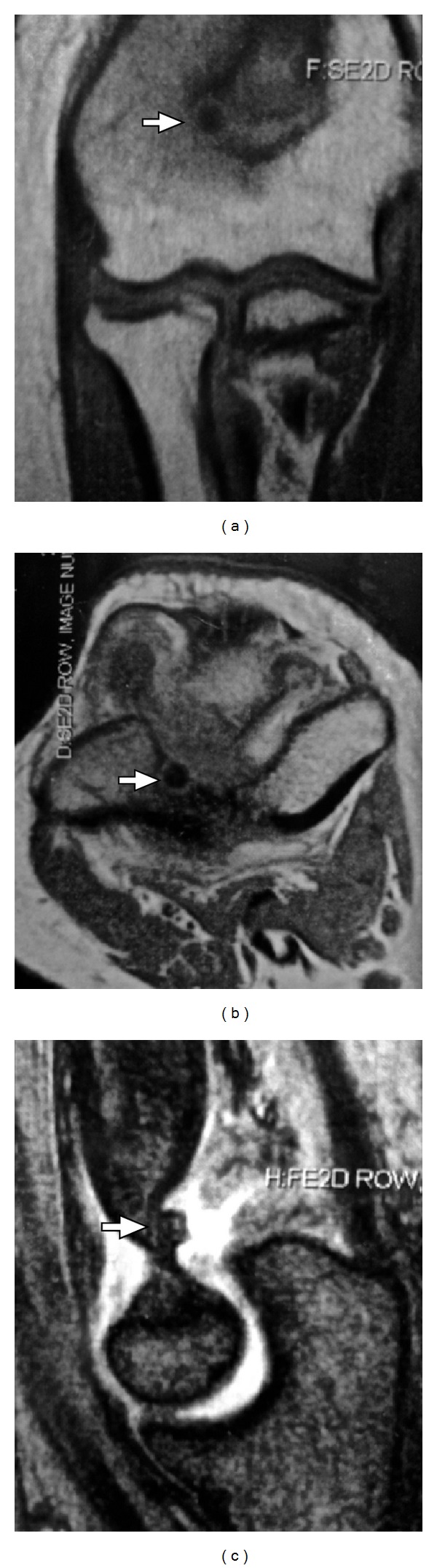
Magnetic resonance image of the nidus located subchondrally and a marked sclerotization of the surrounding bone. No cortical lesion indicating the presence of OO. Signs of mild synovitis were present.

**Figure 5:**
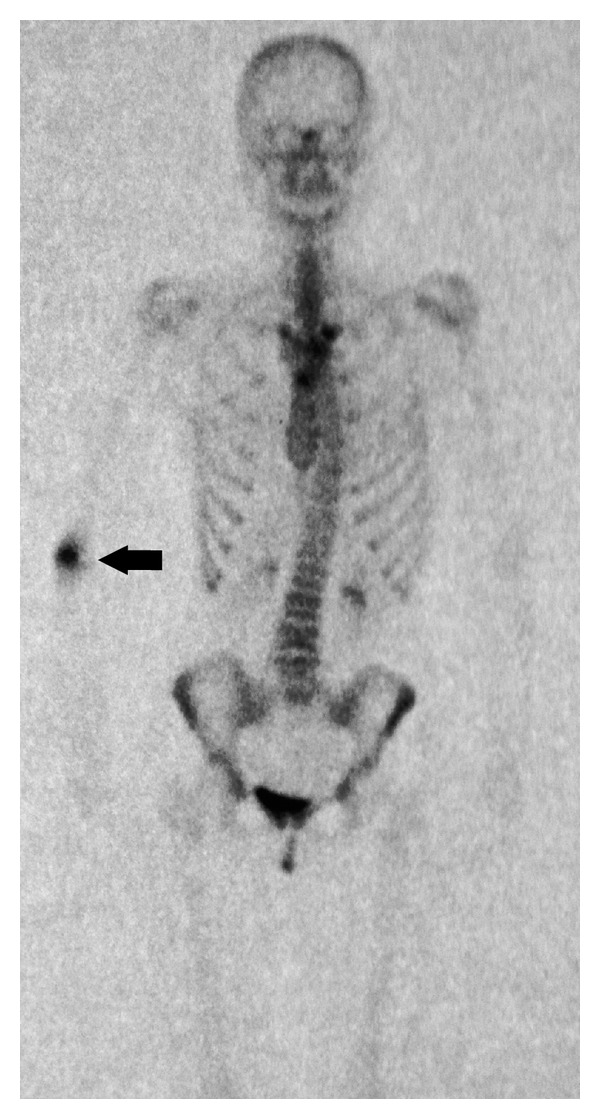
Technetium-99 m bone scan: clearly visible “hot spot” in the distal humerus.

In the radiological evaluation, bony consolidation with correct alignment was observed. A 20-degree Bauman's angle was measured on the coronal plane, and on the sagittal plane, the humerocapitellar angle was 40 degrees. Also, the radiographic study showed a round sclerotic focus on the lateral aspect of the olecranon fossa of about 7 mm in diameter (Figure 1), which was at first interpreted as postfracture bone changes. Nonetheless, this particular radiological finding triggered a battery of tests which comprised: firstly, a scintigraphy in order to study any other possible metastatic lesions, a CT scan for a more precise study of the osseous lesion, and, finally, an MRI to assess the state of articular cartilage due to the lesions proximity to intra-articular structures. The CT scan confirmed the presence of a 7 mm diameter cystic lesion with a central radiolucent nidus that contained a calcified centre (Figure 2). The cystic lesion bulged slightly through the cortical bone and was located subchondrally on the lateral aspect of the olecranon fossa (Figure 3). Magnetic resonance imaging showed that the nidus was located subchondrally with marked sclerotization of the surrounding bone. Signs of mild synovitis were present (Figure 4). The bone scan showed focal uptake of radioisotope corresponding with the site of radiographic abnormality with no other focus (Figure 5). Evaluation of previous and current radiographs, in addition to the CT and bone scan findings, suggested an osteoid osteoma with a rare subchondral localization of the nidus in the depth of the articular surface of the distal humerus.

**Figure 6:**
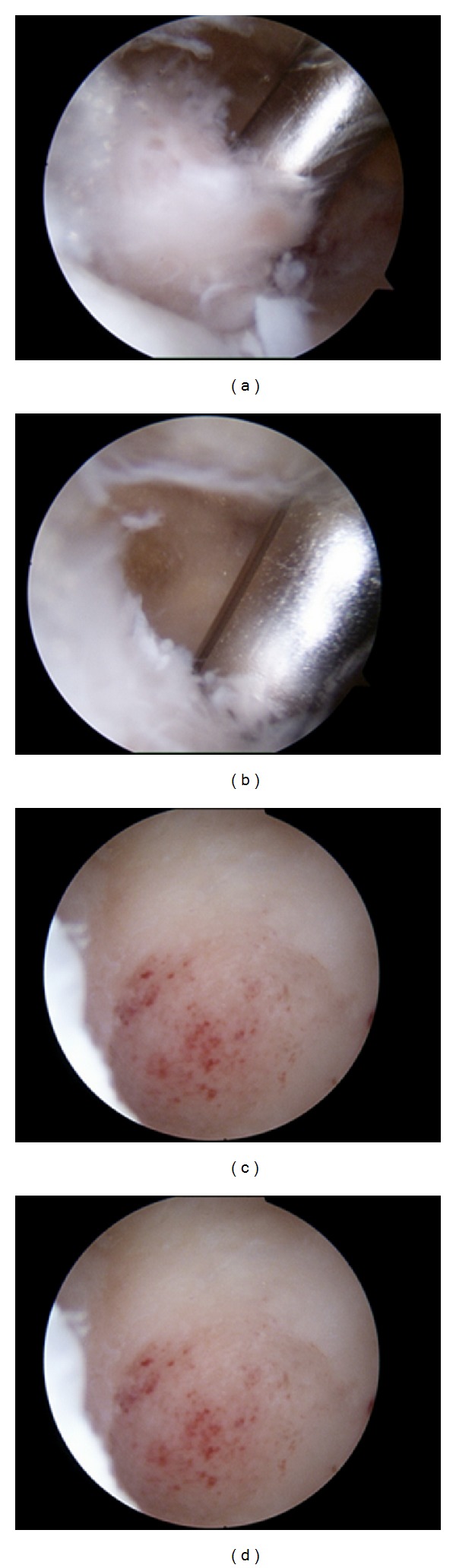
(a) Arthroscopic removal of juxtaarticular osteoid osteoma of the olecranon fossa. (b) Removal of the hypertrophic synovium. ((c) and (d)) The crater at the site of the lesion after its total removal.

Arthroscopic treatment was performed with the patient under general anaesthesia in a lateral decubitus position. A direct lateral approach through the soft point was used for scope insertion. A bony fragment was removed with the curette through an outside-created portal and was sent for pathologic analysis. The lesion, which appeared macroscopically as an elevated bony protuberance with limited surrounding synovitis, was removed with an arthroscopic bone shaver through a medial approach. Hyperaemic and hypertrophied residual tissues were clearly identified and were removed completely without capsular release (Figure 6). Histologic examination confirmed the diagnosis of OO.

Five days after surgery, the patient reported complete pain relief and had regained previous range of motion in the elbow joint. Six weeks of physiotherapy followed. At the 2-year followup, the patient had no clinical or radiological findings that may suggest a recurrence of the tumour.

## 3. Discussion

Juxta- and intra-articular osteoid osteomas are rare, especially in the elbow joint, and usually present an atypical clinical picture mimicking more common entities such as monoarticular arthritis, epicondylitis, synovial, traumatic, or degenerative joint pain [2, 6, 10–16]. The classically described symptoms for the OO are usually altered and may be misleading, especially if the tumour is intra-articular [4]. The pain differs from the classical description in that it is described as less severe and the response to salicylates is less effective [2, 6, 10, 11, 14]. The leading symptoms usually compile joint effusion, synovitis, decreased range of motion and contractures [6, 9, 16]. In our case, the clinical symptoms and radiographic findings were unspecific and had an adding confounding factor: the supracondylar fracture. Such unspecific symptoms led to a delay in the diagnosis given that supracondylar fractures may result in joint contractures, secondary degeneration of articular cartilage, and, more seldom, to growth disturbances and limb deformities in adolescents [6, 10, 17]. Therefore, late diagnosis may lead to symptom aggravation confounding even further the clinical picture [3, 18]. 

Radiologically, as much as one-fourth of all OO are not detected by simple radiographs [6, 19]. In the case at hand, the CT scan did show a sclerotic halo but it lacked the intensity it presents when the lesion is intracortical.

The intensity of the characteristic sclerosis around the nidus has been claimed to vary depending on the anatomical location of the OO; it is more intense in the diaphysis of a long bone and becomes milder in the trabecular bone found in the epimetaphyseal substance [6, 19, 20]. A double ring sign in the bone scintigraphy is characteristic of this lesion; nonetheless, such imaging technique is usually too unspecific to be used by itself for the diagnosis [6, 21–23]. MRI imaging, although described as useful in some circumstances (i.e., in identifying coexistent synovial proliferation and joint effusion), it is usually considered inferior to CT scan in revealing the nidus [11, 19, 23, 24].

When facing an osteoid osteoma, medical and/or surgical treatment may be attempted. Medical prostaglandin suppression with NSAID's has proven successful, nevertheless the average duration of the treatment is of 2-3 years [25]. When opting for surgical treatment, total excision is considered the gold standard and it is what should be attempted in every case. Operative complete excision is therefore the recommended treatment for patients with juxta- or intra-articular osteoid osteomas and may be done openly, percutaneously or arthroscopically [16]. Even though good results are apparently obtained by percutaneous CT-guided excision, drilled resection, and/or radiofrequency coagulation [26], it should be noted that of these techniques, arthroscopic treatment allows for a more thorough histological examination since better visibility of the lesion is obtained [27]. Furthermore, a percutaneous destruction of the lesion with the use of a laser or radiofrequency has been shown to be repeatedly effective with a 91% rate of success [13]; nonetheless, this technique presents higher risk of bone necrosis and soft tissue damage, especially in an anterior elbow joint localization of the tumour [9, 26, 27].

Arthroscopic excision of an intra-articular osteoid osteoma was first reported on the knee [28], since then, arthroscopic treatment of OO has been described in many other joints. This technique presents several advantages when compared to open excisions, a few of them being as follows: reduced postop pain, fewer wound problems, wider intraoperatory vision, less invasive surgery resulting in practically no damage to muscle and ligaments, a shorter rehabilitation period, and a faster return to full activity [6, 9]. The removal of a bony fragment with a curette prior to the total excision may ensure histopathological evaluation [6, 9, 12].

## 4. Conclusion

As shown in this paper, the diagnosis of an intra-articular osteoid osteoma in the elbow joint can be confounded due to atypical clinical symptoms and radiological findings. When a confounding factor, such as a previous trauma, is added to the equation, it may easily lead to misdiagnosis of an OO mistaking it as enthesopathy or synovial disease, delaying diagnosis and treatment. Complete excision of the lesion must be made, and an arthroscopic approach may offer several advantages over other more aggressive techniques.
